# Recent Advances in ^18^F-Labeled Amino Acids Synthesis and Application

**DOI:** 10.3390/pharmaceutics14102207

**Published:** 2022-10-17

**Authors:** Chao Wang, Rong Lin, Shaobo Yao

**Affiliations:** Department of Nuclear Medicine, The First Affiliated Hospital of Fujian Medical University, No. 20 Chazhong Road, Fuzhou 350005, China

**Keywords:** fluorine-18, amino acids, positron emission tomography, radiopharmaceuticals, radiofluorination

## Abstract

Radiolabeled amino acids are an important class of agents for positron emission tomography imaging that target amino acid transporters in many tumor types. Traditional ^18^F-labeled amino acid synthesis strategies are always based on nucleophilic aromatic substitution reactions with multistep radiosynthesis and low radiochemical yields. In recent years, new ^18^F-labeling methodologies such as metal-catalyzed radiofluorination and heteroatom (B, P, S, Si, etc.)-^18^F bond formation are being effectively used to synthesize radiopharmaceuticals. This review focuses on recent advances in the synthesis, radiolabeling, and application of a series of ^18^F-labeled amino acid analogs using new ^18^F-labeling strategies.

## 1. Introduction

Positron emission tomography (PET) is attracting considerable attention in medical imaging technology. It can provide noninvasive, functional, and metabolic information at the molecular level and plays an increasingly important role in the diagnosis and staging of tumors [1]. As a commonly used tracer for PET imaging, 2-[^18^F]fluoro-2-deoxy-D-glucose ([^18^F]FDG) can provide valuable functional information based on the increased glucose uptake of cancer cells and can describe metabolic abnormalities. [^18^F]FDG PET/CT is more sensitive and specific in certain cancers, such as lymphoma, non-small cell lung cancer, and esophageal cancer, and can be used for tumor staging and restaging. It has also been used to assess treatment response, which can distinguish responders from non-responders before morphological alterations occur [2,3,4]. However, due to high uptake in the normal brain, [^18^F]FDG is met with limited success in acquiring images with adequate contrast in brain tumors [5]. Moreover, some types of tumors may transform their metabolic consumption from glucose to other nutrients such as amino acids (AAs) [6,7].

AAs can be actively transported to the brain and be used to visualize brain tumors that are not associated with the disruption of the blood–brain barrier. Malignant tumors are associated with rapid growth and unrestricted cell proliferation, which require the uptake and consumption of high levels of AAs [8,9]. Therefore, certain AA transporters (AATs) are overexpressed or upregulated in tumor cells as carriers for the transport of AAs into cells [10,11,12]. The mechanism of AA uptake by tumor cells during molecular imaging mainly reflects AAT activity rather than protein synthesis; moreover, several unnatural AAs do not participate in protein synthesis but are a part of AATs. Therefore, the imaging of positron-labeled AAs in patients with tumors can reflect the expression of AATs in tumor cells [13].

The advantageous nuclear-decay properties of fluorine-18 (97% β^+^, 109.7 min half-life) permit its use in multistep synthesis, thereby rendering it an ideal radionuclide for the radiolabeling of molecules as potential PET tracers. Over the past decades, several aromatic AAs such as phenylalanine (Phe) and tyrosine (Tyr) have been radiolabeled with fluorine-18 via nucleophilic aromatic substitution (S_N_Ar) for PET imaging [14,15,16,17]. In S_N_Ar reactions with [^18^F]fluoride, a leaving group (-N^+^Me_3_, -NO_2_, -halogens, -mesylate, etc.) and an activating group (-NO_2_, -CN, -CF_3_, or -carbonyl groups, etc.) in the ortho or para position, with respect to the leaving group, is usually necessary in the precursor, which limits the substrate scope. Moreover, amino, hydroxyl, and other active groups in AAs will interfere with the S_N_Ar reaction; thus, protection and deprotection steps are usually required, which may prolong synthesis time. Therefore, new synthesis strategies are always needed for the ^18^F-labeling of AAs to expand substrate scope and shorten synthesis time.

In the past decade, significant progress has been made in the field of labeling methods via the direct ^18^F-fluorination of nonactivated arenes and aliphatic carbon compounds. Among them, hypervalent iodine (III) methods and copper-mediated strategies using pinacol boronate (BPin) ester or boronic acid precursors are currently the most commonly used methods. Light-mediated radiofluorination strategies allow for the formation of ^18^F-labeled compounds at room temperature and are known to have good functional group tolerance. Fluoride bond formation in heteroatoms such as B-^18^F, Si-^18^F, and P-^18^F is possible at room temperature using ^18^F-labeling in aqueous media [18,19,20]. These methods have also been used for the synthesis of ^18^F-labeled AA analogs. In this review, we focus on the new strategies used for the synthesis of ^18^F-labeled AA analogs and their uses.

## 2. Metal-Free ^18^F-AA Synthesis

**Figure 1:**
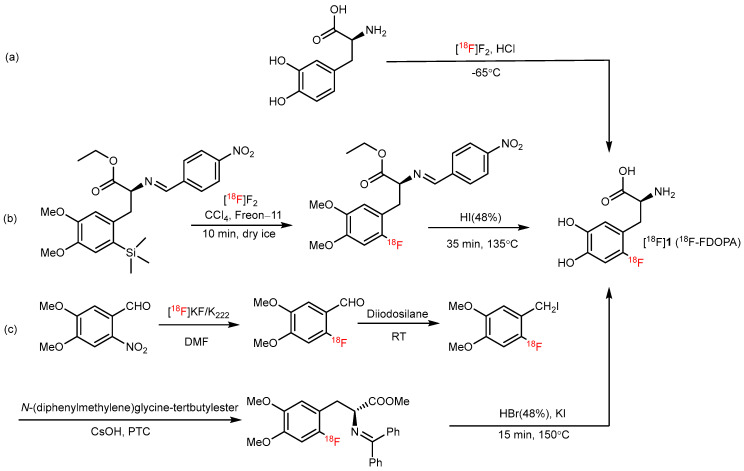
(**a**) Synthesis of [^18^F]**1** through [^18^F]F_2_. Modified from Firnau et al. [22]. (**b**) Electrophilic synthesis of [^18^F]**1**. Modified from Diksic et al. [23]. (**c**) Synthesis of [^18^F]**1** through an asymmetric alkylation by Shen et al. [27].

Since the 1970s, AAs have been labeled with fluorine-18 owing to the important role of AAs in disease progression [21]. For example, 6-[^18^F]F-DOPA ([^18^F]**1**), an AA analog PET tracer used to image the presynaptic dopaminergic system in the brain, was first radiosynthesized with [^18^F]F_2_ in 1984; however, its low radiochemical yield (RCY; 3%) limits its use in routine production (Figure 1a) [22]. A desilication reaction has also been used to synthesize [^18^F]**1** to obtain high regioselectivity and good RCY; however, manipulating [^18^F]F_2_ is complex, and it is challenging to obtain high molar activity (A_m_) [^18^F]**1** via an electrophilic reaction (Figure 1b) [23]. Although many researchers have begun to synthesize [^18^F]**1** using nucleophilic substitution strategies, multistep radiosynthesis is complicated in an automated module (Figure 1c) [24,25,26,27]. Therefore, the convenient and automated synthesis of [^18^F]**1** continues to pose a challenge.

## 3. Copper-Catalyzed ^18^F-AA Synthesis

Direct radiofluorination on AAs bearing electron-rich arenes, such as Phe, Tyr, and tryptophan (Trp), is a difficult task when traditional S_N_2 or S_N_Ar reactions are used. To solve this problem, several new fluorine-18 radiochemical methodologies have been reported [28,29,30,31]. As transition-metal catalysis can accelerate radiofluorination reaction rates and enhance selectivity and reactivity, transition metal-catalyzed methodologies have emerged as an attractive technique with which to fluorinate arenes with nucleophilic fluoride sources [32,33]. The cationic Pd(IV) complex, Ni−Aryl complex, diaryliodonium salts, and spirocyclic iodonium ylides (SCIDY) structure, etc., have been studied for the fluorination of electron-rich arenes and have also been used for AA radiofluorination.

**Figure 2:**
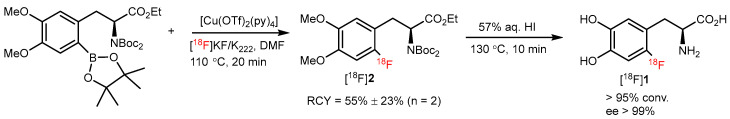
Radiosynthesis of [^18^F]**1** via Cu-mediated ^18^F-fluorination of aryl boronate ester precursor. Modified from Tredwell et al. [35].

Cu-mediated radiofluorination (CMRF), particularly, has emerged as a powerful strategy for constructing C–^18^F bonds, and BPin esters and boronic acids were the most popular substrates to label aromatic AAs using CMRF [34]. In 2014, the Gouverneur group reported a CMRF reaction of pinacol-derived aryl boronic esters with a diverse range of substrates. Using CMRF, [^18^F]**1** was successfully synthesized with high RCY from an arylBPin precursor (Figure 2). In a typical run of the automated synthesis of [^18^F]**1**, a dose of 609 MBq of the product was isolated from a starting material of 13 GBq [^18^F]fluoride, which was equal to a 12% decay corrected RCY [35].

**Figure 3:**
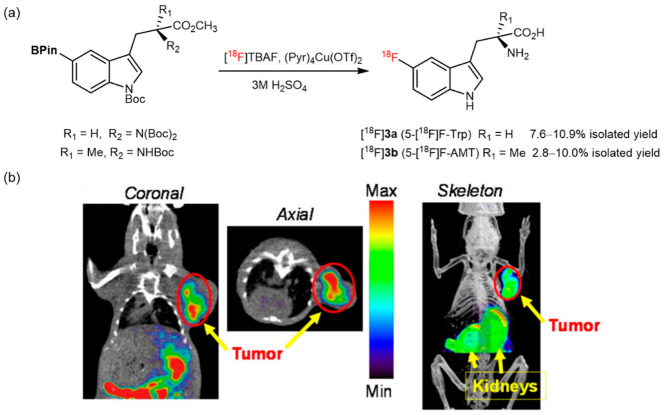
(**a**) Synthetic route of [^18^F]**3a** and [^18^F]**3b**. Modified from Giglio et al. [38]. (**b**) Decay corrected whole-body microPET-CT images of [^18^F]**3b** in B16F10 melanoma xenografts 30 min postinjection. Reprinted with permission from ref. [38], Copyright Ivyspring International Publisher.

Tryptophan plays an important role in metabolism in multiple diseases and can especially indicate the expression of indoleamine 2,3-dioxygenase (IDO1), an enzyme that maintains normal tryptophan homeostasis [36,37]. Considering the significance of IDO1 in cell proliferation and cancer-cell growth, Giglio and coauthors prepared [^18^F]**3a** and [^18^F]**3b** using a similar CMRF reaction to monitor IDO1 activity in vivo (Figure 3a). [^18^F]**3b** accumulation is associated with IDO1 expression in HeLa cells. The micro PET-CT imaging of [^18^F]**3b** showed obvious tumor uptake in the B16F10 melanoma model (Figure 3b); however, the uptake of [^18^F]**3a** did not correlate with IDO1 activity in vivo, which may be attributed to the metabolism of the radiotracer [38]. However, this reaction is sensitive to bases and requires oxygen, thereby posing a challenge to modern automation modules [39].

**Figure 4:**
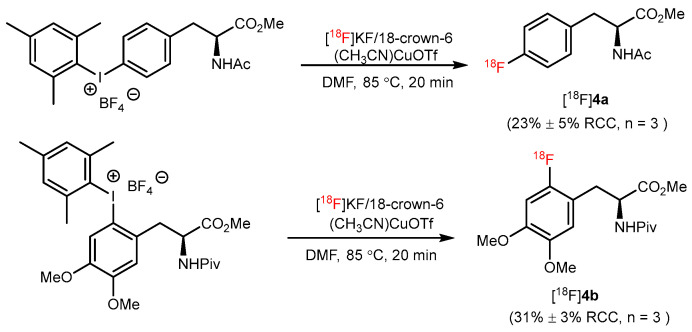
Synthesis of [^18^F]**4a** and [^18^F]**4b** through (mesityl)(aryl)iodonium salts. Modified from Ichiishi et al. [32].

The CMRF of aryl(mesityl)iodonium salts is also highly attractive for some merits, including mild reaction conditions, high regioselectivity, etc. [40]. In 2014, Sanford and Scott reported a CMRF of (mesityl)(aryl)iodonium salts using [^18^F]KF with diverse aromatic substrates. Two different AA-derived substrates ([^18^F]**4a** and [^18^F]**4b**) were synthesized in mild conditions with moderate yields. Additionally, [^18^F]**4b** was radiosynthesized using an automated synthesis module with high Am (4 ± 2 Ci/µmol) (Figure 4) [32].

**Figure 5:**
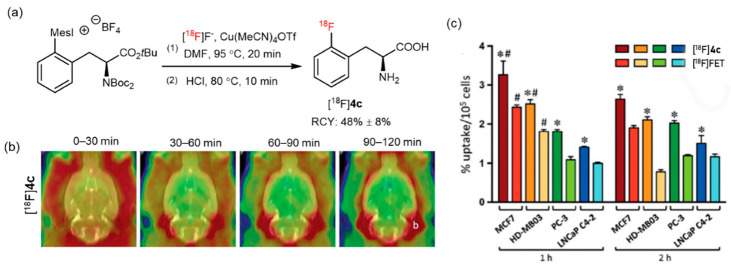
(**a**) Radiosynthesis of [^18^F]**4c** from an iodonium salt precursor. Modified from Modemann et al. [41]. (**b**) Biodistribution of [^18^F]**4c** in healthy rat brains. (**c**) Absolute uptake of [^18^F]**4c** and [^18^F]FET in different tumor cell lines. * [^18^F]**4c** uptake was significantly higher than [^18^F]FET uptake. F(7, 32) = 185.9, *p* < 0.0001, Sidak’s post-hoc *p* < 0.05. # Tracer uptake was significantly higher after 1 h compared to 2 h of incubation. F(1, 32) = 53.9, *p* < 0.0001; Sidak’s post-hoc *p* < 0.05. Reprinted with permission from ref. [41], Copyright 2019, Georg Thieme Verlag KG.

Another imaging agent [^18^F]**4c** (2-[^18^F]FPhe) was also synthesized using this protocol using “low base” or “minimalist” conditions (Figure 5a). The stability of [^18^F]**4c** toward defluorination was studied in healthy rat brains and was found to have sufficient in vivo stability (Figure 5b). Furthermore, initial biological studies revealed a higher uptake of [^18^F]**4c** in different tumor cells compared with that of [^18^F]FET (Figure 5c). Thus, [^18^F]**4c** is a promising PET probe for further research [41]. However, this approach also has some limitations; (mesityl)(aryl)iodonium salts are challenging to synthesize and can have a limited shelf life.

**Figure 6:**
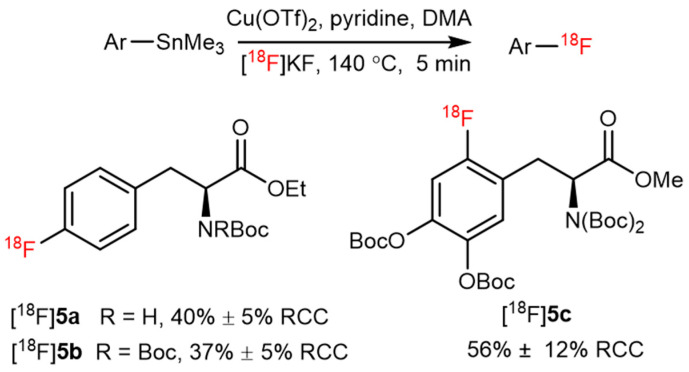
Radiosynthesis of [^18^F]**5a**,**5b** and [^18^F]**5c** from trimethyl(phenyl)tin precursor. Modified from Makaravage et al. [42].

Trimethyl(phenyl)tin is another attractive precursor for C–[^18^F]F bond formation owing to its good reactivity and stability during CMRF. In 2016, the first practical nucleophilic fluorination of stannanes using ^18^F was reported by Scott et al. The CMRF reaction is compatible with both electron-deficient and electron-rich arene substrates. Clinically relevant radiotracers such as [^18^F]**5a**,**5b** (protected phenylalanine derivatives) and [^18^F]**5c** (protected [^18^F]**1**) were obtained with high radiochemical conversion (RCC) using this approach (Figure 6) [42].

**Figure 7:**
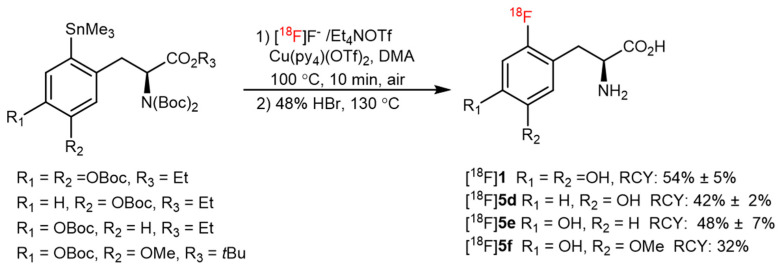
The preparation of [^18^F]**1** and [^18^F]**5d**–**5f** from a Cu-mediated ^18^F-fluorination of arylstannanes. Modified from Zarrad et al. [43].

Zarrad and coworkers also evaluated a CMRF reaction using trimethyl(phenyl)tin as a model substrate to label aromatic AAs. [^18^F]**1** and [^18^F]**5d**–**5f** were synthesized in two steps using automation module on a preparative scale with isolated RCYs of 32–54% (Figure 7) [43].

**Figure 8:**
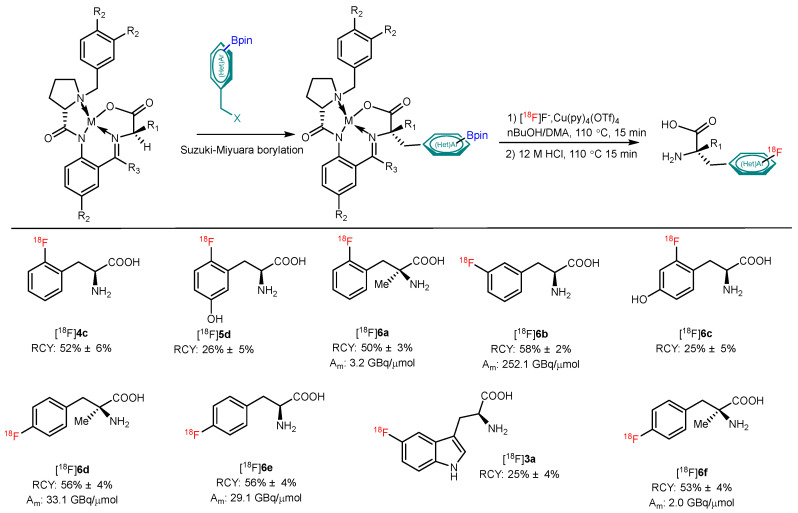
Application of Ni/Cu-BPX templates for the late-stage preparation of ^18^F-labeled AAs. Modified from Craig et al. [44].

In 2020, Craig and coworkers reported an alcohol-enhanced CMRF reaction of BPin-substituted Ni–BPX–AAA complexes for the synthesis of diverse radiolabeled AAs (Figure 8). Convenient precursor synthesis, high RCYs, and accessibility to automation radiolabeling may render this method more practical [44].

## 4. Ruthenium-Catalyzed ^18^F-AA Synthesis

**Figure 9:**
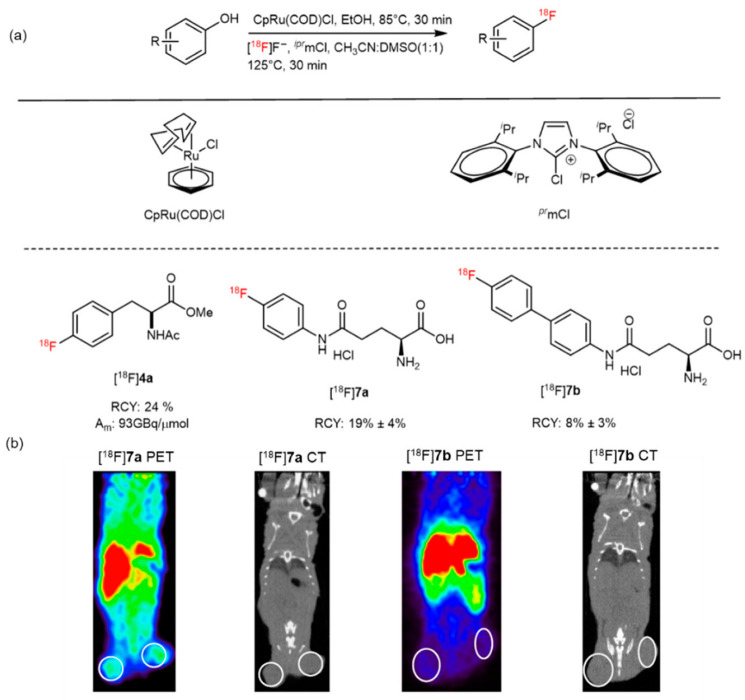
(**a**) ^18^F-fluorophenylalanine derivatives, [^18^F]**7a** and [^18^F]7**b,** were synthesized from an *^iPr^*ImCl-mediated deoxyfluorination reaction. Modified from Beyzavi et al. [45]. (**b**) Micro PET and CT images of [^18^F]**7a** and [^18^F]**7b** in PC-3 xenografts of a tumor-bearing mouse. Reproduced from ref. [46], Copyright 2020, with permission from Elsevier.

In 2017, Ritter et al. reported a direct aromatic fluorination method using the ruthenium *π*-complex, and [^18^F]**4a** was auto-synthesized within 80 min in a 24% isolated RCY (Figure 9a) [45]. In another study, two glutamine-derived PET tracers, [^18^F]**7a** ([^18^F]fluorophenylglutamine) and [^18^F]**7b** ([^18^F]fluorobiphenylglutamine), were also labeled and evaluated using the same method; however, both tracers had a low affinity toward the rat ASCT-2 transporter in vitro and low uptake in the F98 rat xenograft in vivo (Figure 9b). The authors concluded that they would optimize the substituents of the arene ring to obtain a high-quality, glutamine-based PET radiotracer [46].

## 5. Manganese-Catalyzed ^18^F-AA Synthesis

**Figure 10:**
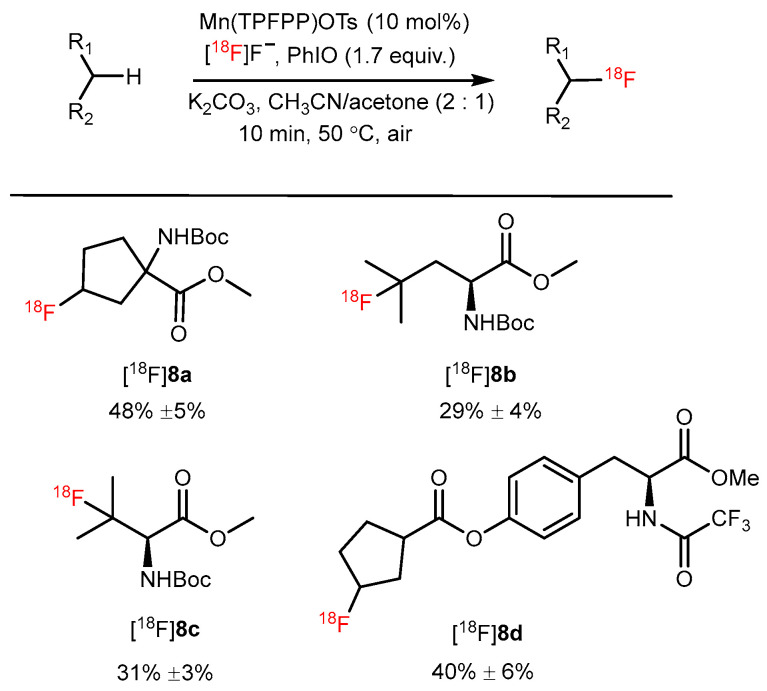
Several protected amino acid analogs were prepared by a Mn-mediated aliphatic C–H ^18^F fluorination reaction. Modified from Liu et al. [47].

Groves and coworkers developed a manganese porphyrin-mediated ^18^F-labeling method that could selectively fluorinate inactivated aliphatic C–H bonds with no carrier-added [^18^F]fluoride. Several protected AA analogs ([^18^F]**8a**–**8d**) were ^18^F-labeled in one step using this method, with the RCC ranging from 12% to 67% (Figure 10) [47].

In summary, transition metal-mediated radiofluorination is an attractive strategy for the one-step radiosynthesis of AAs bearing electron-rich arenes. However, potential metal contamination is a nonnegligible problem to be considered when this strategy becomes commonly used in clinical.

## 6. Photocatalyzed ^18^F-AA Synthesis

**Figure 11:**
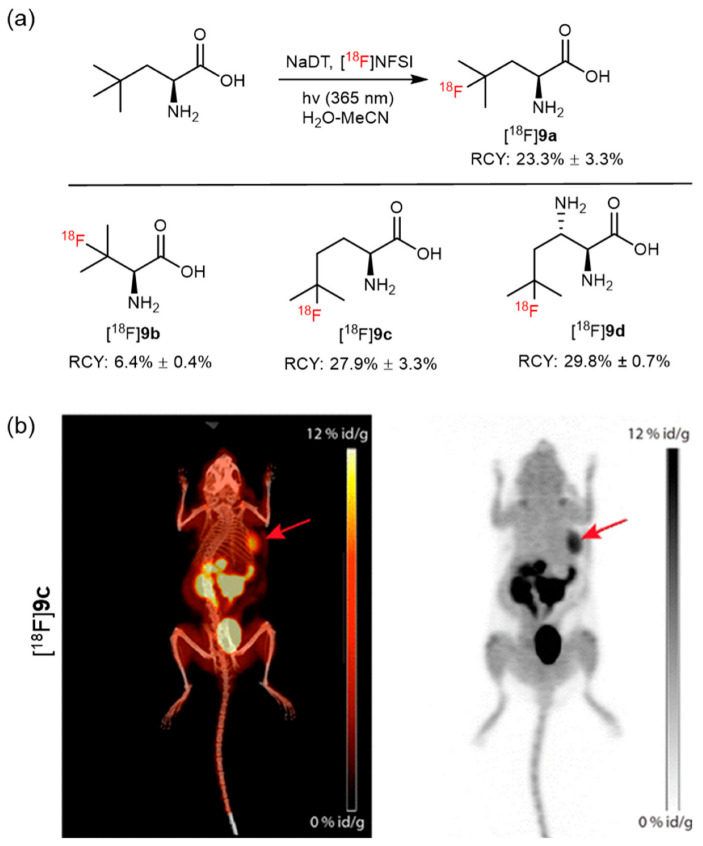
(**a**) Synthesis of [^18^F]**9a**–**9d** via a photocatalytic reaction. Modified from Nodwell et al. [50]. (**b**) PET images of compound [^18^F]**9c** in xenografted mice with human glioma. Maximum intensity projection images overlaid on CT and standalone PET images of the biodistribution of [^18^F]**9c** at 60 min show high accumulation of [^18^F]**9c** in the tumor (red arrow). Reproduced from ref. [50], Copyright 2017, American Chemical Society.

Compared with traditional nucleophilic aromatic ^18^F-fluorination, photo-mediated radiofluorination is associated with milder reaction conditions and high functional group tolerance and could be used for electron-rich arenes [48]. Britton et al. reported a UV light-promoted, C–H-selective fluorination of aliphatic and benzylic substrates using the HAT photocatalyst tetrabutylammonium decatungstate (TBADT) and N-fluorobenzenesulfonimide as the fluorine atom-transfer reagent [49]. In 2017, this methodology was also used for C–H bond radiofluorination in unprotected and branched aliphatic AAs ([^18^F]**9a**–**9d**) for PET imaging (Figure 11a). No obvious defluorination and high accumulation in human glioma and prostate cancer xenografts were observed during the micro PET-CT imaging of [^18^F]**9c** (Figure 11b), thereby warranting its further use [50].

**Figure 12:**
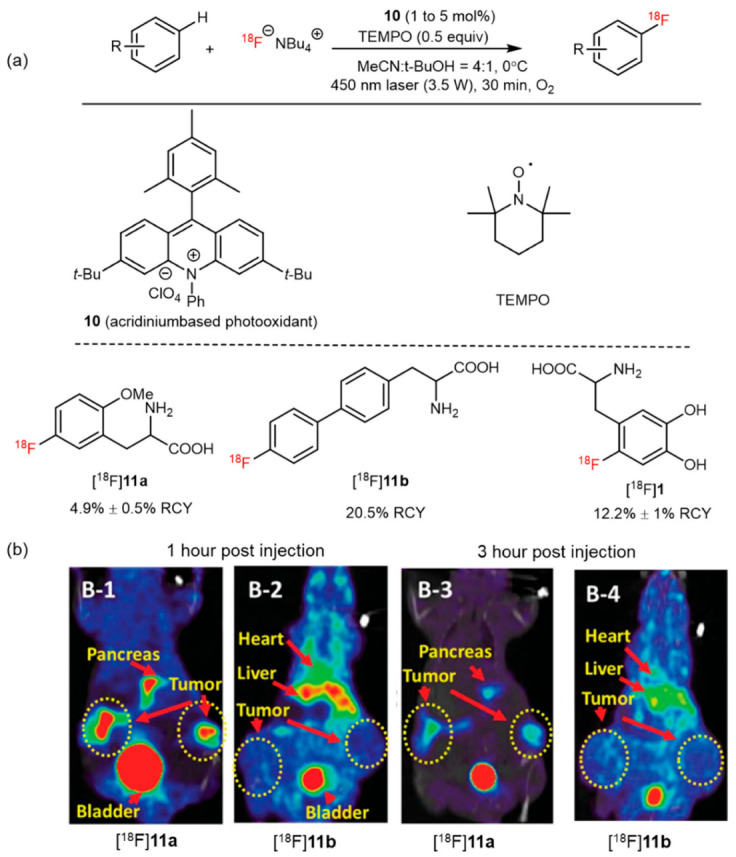
(**a**) Several amino acid analogs prepared by a photoredox radiolabeled fluorination of C(sp^2^)-H. Modified from Chen et al. [51]. (**b**) PET-CT images of [^18^F]**11a** and [^18^F]**11b** in mice containing MCF7 (breast cancer) xenografts. Reproduced from ref. [51], Copyright 2019, The American Association for the Advancement of Science.

In 2019, Li and coworkers reported a C–H ^18^F-fluorination method in several arene substrates using photoredox catalysts (**10**). Under illumination of a 3.5-W laser (450 nm) in a mixed system of 4:1 MeCN:*t*-BuOH with O_2_ as an oxidant, TEMPO as a redox cocatalyst, and [^18^F]F^−^/TBAF as a fluoride source, [^18^F]**1** and [^18^F]**11a**,**11b** were successfully labeled and obtained with good RCY (Figure 12a). Furthermore, in vivo PET studies of compound [^18^F]**11a** revealed higher tumor accumulation in human breast cancer (MCF-7)-bearing mice compared with compound [^18^F]**11b** that exhibited nonspecific binding (Figure 12b). These findings demonstrate the potential of radiofluorination in the design and synthesis of novel PET agents [51].

**Figure 13:**
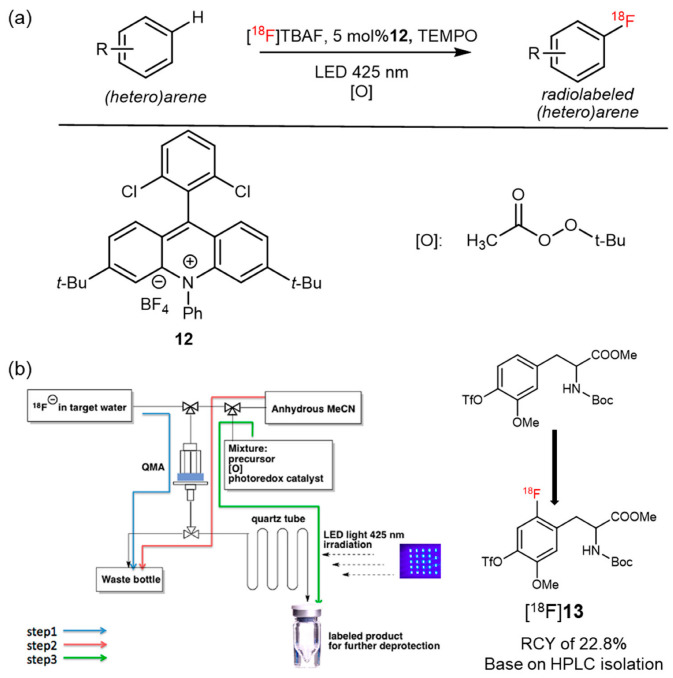
(**a**) Photo-mediated radiofluorination reaction. Modified from Wang et al. [52]. (**b**) Schematic workflow of preparing ^18^F-Labeled agent and its application in synthesis [^18^F]**13**. Reproduced from ref. [52], Copyright 2017, American Chemical Society.

In 2020, Nicewicz, Li, and coworkers further improved their previously reported methodology by replacing laser irradiation with a blue light-emitting diode (LED) source and by using an organic oxidant *tert*-butyl peroxyacetate rather than oxygen. Moreover, a library containing 48 organic photocatalysts was evaluated in this study, and compound **12** was determined to be the most suitable catalyst for radiofluorination (Figure 13a). This procedure is suitable to perform in a microfluidic reactor and continues to retain the advantages reported in their previous study. [^18^F]**13** (protected [^18^F]**1**) was also synthesized using this method with a 22.8% isolated RCY (Figure 13b). This simplified procedure may be used as a general methodology for the synthesis of novel ^18^F-labeled radiotracers [52].

**Figure 14:**
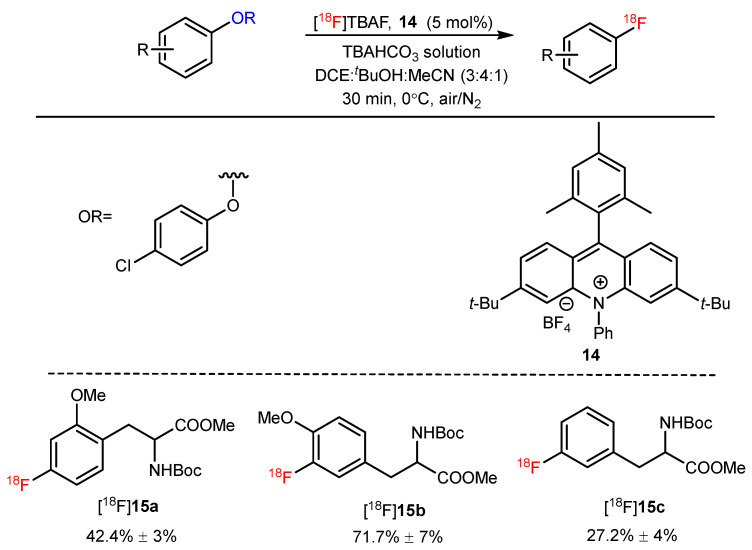
Synthesis of [^18^F]**15a**–**15c** through a photoredox-catalyzed nucleophilic deoxyfluorination reaction. Modified from Tay et al. [53].

Recently, Nicewicz and Li et al. reported the photoredox-catalyzed nucleophilic deoxyfluorination reaction of phenol derivatives. In this method, TBAHCO_3_ was used as a phase-transfer agent and an acridinium-based photo oxidant (**14**) was used under 34-W blue LED irradiation to introduce fluorine-18 into electron-rich arenes. N-Boc-O-methyltyrosines and -phenylalanine ([^18^F]**15a**–**15c**) were also successfully radiofluorinated (Figure 14) [53].

While obvious advances in the light-mediated formation of C-^18^F bonds have been explored in recent years, several limitations still remain, including the lack of essential equipment for the preparation of ^18^F-labeled compounds and the reproducibility of experiments in other laboratories.

## 7. Synthesis of ^18^F-Labeled AAs via [^18^F]Trifluoromethylation Reaction

**Figure 15:**
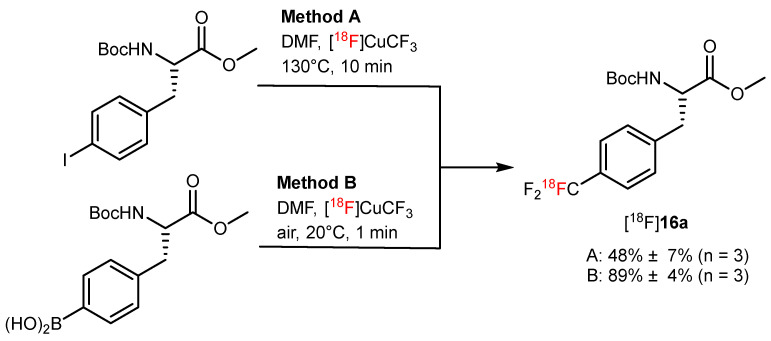
Synthesis of [^18^F]**16a** via a [^18^F]trifluoromethylation reaction. Modified from van der Born et al. [56].

As an excellent bioisostere of the methyl group, trifluoromethyl (CF_3_) is a common functional group during the synthesis of pharmaceuticals. An increasing number of [^18^F]trifluoromethylation labeling methods have been reported and used for the radiosynthesis of AA analogs [10,54,55]. Dion et al. set [^18^F]CuCF_3_ as the radiofluorination agent and boronic acids or iodides as a leaving group to successfully synthesize Boc/OMe-protected 4-[^18^F]trifluoromethylphenylalanine ([^18^F]**16a**) in high RCY up to 89% using two different strategies (Figure 15) [56].

**Figure 16:**
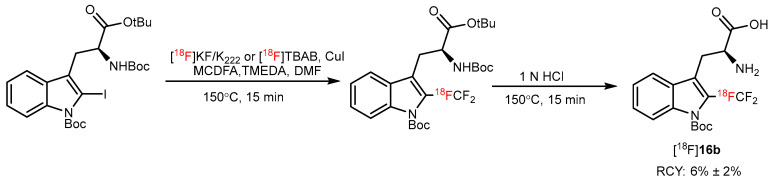
Radiosynthesis of [^18^F]**16b** by a Cu(I)-mediated [^18^F]trifluoromethylation reaction. Modified from Kim et al. [57].

In 2019, Kim et al. reported a Cu(I)-mediated [^18^F]trifluoromethylation method to synthesize [^18^F]trifluoromethyl-L-tryptophan ([^18^F]**15b**); however, the radiochemical yield of this tracer was 6%, and molar activity was 0.76 GBq/μmol (Figure 16) [57].

**Figure 17:**
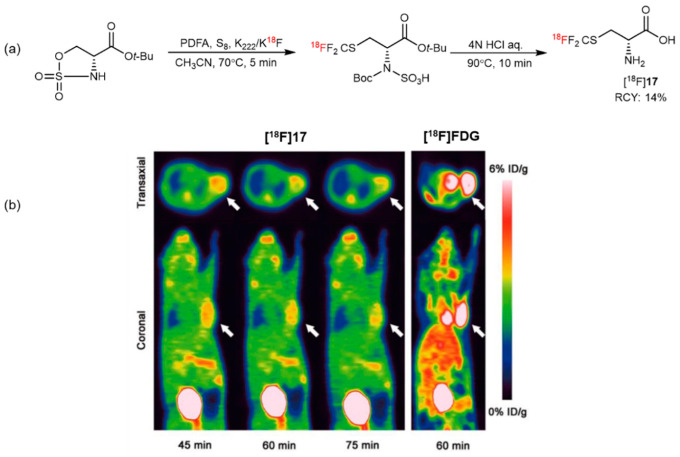
(**a**) [^18^F]Trifluoromethyl cysteine synthesis from cyclic sulfamidates. Modified from Liu et al. [58]. (**b**) PET images of C6 glioma-bearing mice were scanned at 45, 60, and 75 min after injection of [^18^F]**17** and 60 min after injection of [^18^F]FDG (the white arrow indicates the tumor). Reprinted with permission from ref. [58], Copyright 2019, Georg Thieme Verlag KG.

Using structure-based bioisosterism, Tang et al. synthesized ^18^F-trifluoromethylated cysteine enantiomers as “structure-mimetic” AA tracers for glioma PET imaging (Figure 17a). Meanwhile, compared with [^18^F]FDG, *S*-[^18^F]CF_3_-D-Cys ([^18^F]**17**) exhibited much lower uptake in most organs, especially in brain tissue, and a 3.81 ± 0.23% ID/g tumor uptake was reported 45 min after injection (Figure 17b) [58]. In another study, Tang and coworkers further explored the potential application of [^18^F]**17** in evaluating glioma by comparing magnetic resonance imaging (MRI) and histopathology. The results showed that, compared with [^18^F]FDG and [^18^F]**1**, [^18^F]**17** had the highest TNRs in the same orthotopic C6 glioma models, suggesting that the tracer may serve as a valuable tool in the diagnosis of gliomas [59].

## 8. Synthesis of ^18^F-Labeled AAs via B-^18^F Bond Formation

**Figure 18:**
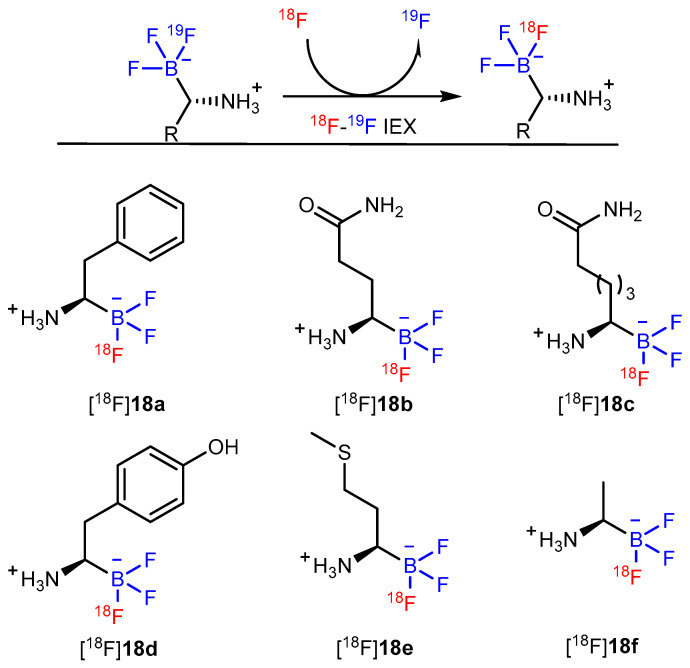
Synthetic route and several examples of ^18^F-BAAs. Modified from Liu et al. [63].

**Figure 19:**
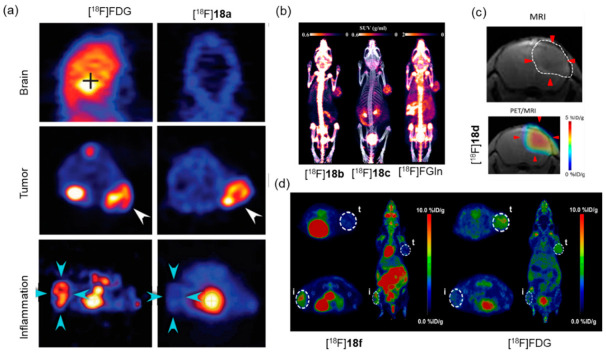
(**a**) [^18^F]**18a** shows specific accumulation in U87MG xenografts and low uptake in normal brain and an inflammatory region. Reproduced from ref. [63], Copyright 2019, The American Association for the Advancement of Science. Normal brain tissue indicated by “+”, tumor regions indicated by white arrows, and inflammatory regions indicated by cyan arrows. (**b**) Representative small-animal PET/CT imaging of [^18^F]**18b**, [^18^F]**18c**, and [^18^F]FGln in the same BGC823 xenograft-bearing mice at 45 min post-injection. Reproduced from ref. [68], Copyright 2017, American Chemical Society. (**c**) Representative contrast-enhanced, T1-weighted MR coronal images and merged [^18^F]**18d** PET/MR images of orthotopic U87glioma. Reprinted from ref. [69], Copyright 2019, with permission from Springer Nature. (**d**) Representative PET imaging of [^18^F]**18f** and [^18^F]FDG in nu/nu mice bearing BGC-823 xenografts (right shoulder) and inflammation model (left hindlimb) at 45 min post-injection. Reproduced from ref. [73], Copyright 2017, American Chemical Society.

After Ting et al. reported two classes of biomolecule precursors (i.e., arylfluoroborates and alkylfluorosilicates) that could afford late-stage, one-step ^18^F-labeling with high stability in aqueous media, the development of the B–F bond gained popularity [60,61,62]. A general method to synthesize trifluoroborate AA derivatives using boramino acids (BAAs) was developed by Liu et al. to simulate natural AAs by demonstrating the biological similarity between the trifluoroborate and carboxylate groups (Figure 18). Furthermore, the ^18^F-^19^F isotope exchange reaction to label [^18^F]-BAA is quite simple, does not require HPLC purification, and provides good radiochemical yields (>60%, non-decay corrected) and molar activity (>37 GBq/mmol). Furthermore, the biological similarity between the trifluoroborate (-BF3^−^) and carboxylate groups (COO^−^) was demonstrated. Cellular assays revealed that the uptake of [^18^F]-BAAs was AAT-mediated cell uptake, whereas in vivo studies showed high tumor-specific accumulation. Almost all AAs can be ^18^F-labeled similarly for imaging AA transporter activity. [^18^F]-Phe-BF_3_ ([^18^F]**18a**), as an analog of Phe, shows specific accumulation in U87MG xenografts. Unlike [^18^F]FDG, its uptake is low in the normal brain and inflamed regions (Figure 19a) [63]. Zhou et al. performed [^18^F]**18a** PET imaging on healthy volunteers. Dynamic imaging revealed that the agent could be distributed and metabolized rapidly, and that uptake by systemic parenchymal organs was low and mainly cleared by the kidneys. Thus, [^18^F]**18a** shows promise as a new imaging agent in a clinical setting owing to its low background interference [64]. A recent study suggests that glutamine (Gln) can be the primary energy source for tumor cells. Several FDG-negative tumors rely on glutaminolysis for energy generation [65,66]. Therefore, [^18^F]FBQ ([^18^F]**18b**) was radiosynthesized and evaluated in a tumor-bearing animal model inoculated with 4T1 xenografts. Small-animal PET imaging showed visible tumor uptake and rapid renal clearance; however, high uptake by the bone indicates the unsuitability of [^18^F]**18b** for clinical translation [67]. [^18^F]FBQ-C_2_ ([^18^F]**18c**) was synthesized to improve the stability of [^18^F]**18b**. Stability studies indicated it to be more stable than [^18^F]**18b** both in vitro and in vivo, and no obvious bone uptake was determined (<1%ID/g). A competitive inhibition assay revealed that [^18^F]**18c** was taken up by cancer cells through the system ASC and N, which is similar to that used by Gln. Moreover, [^18^F]**18c** shows better accumulation in tumors than [^18^F]**18b** and [^18^F]FGln during PET imaging, thereby making it a promising PET tracer for tumor diagnosis [68].

[^18^F]-B-MET ([^18^F]**18d**) was the first ^18^F-labeled, methionine-based tracer to be synthesized and evaluated in three glioma tumor models (C6, GL26, and U87) using PET imaging. The results revealed that LAT-1 is responsible for tracer uptake in brain-tumor models, and the glioma tumor was rapidly detected by the tracer. Moreover, higher [^18^F]**18d** uptake was also found to colocalize with the enhancement in T1-enhanced MRI in an orthotopic U87 human glioma model (Figure 19c). Its favorable biological properties and push-button synthesis make it a potential candidate for clinical translation [69].

[^18^F]**18e** ([^18^F]-FBY) was initially used as a theranostic for imaging-guided boron neutron capture therapy by evaluating the biodistribution of **18e** using mouse tumor models [70]. Six healthy volunteers were injected with [^18^F]**18e** to evaluate the safety and radiation dosimetry. The results showed that [^18^F]**18e** was cleared mainly through the renal system and was well tolerated by all healthy volunteers with no obvious adverse symptoms. Additionally, [^18^F]**18e** was capable of producing an obvious contrast in the glioma tumors of 13 patients with suspected primary or recurrent diffuse gliomas [71]. In another study, a [^18^F]**18e** PET scan was performed on 35 patients with suspected malignant brain tumors for further diagnosis. The study group found that all primary glioblastoma, recurrent glioma, and metastatic brain tumor cells could significantly take up [^18^F]**18e** [72]. Therefore, [^18^F]**18e** shows immense potential in the diagnosis, staging, and prognosis of patients with gliomas. In a follow-up study, an ^18^F-labeled alanine derivative ([^18^F]**18f**) was reported for cancer imaging. Good tumor contrast was achieved in xenograft tumor-bearing mice, and the tracer could distinguish tumors from inflammation in vivo (Figure 19d) [73].

## 9. Synthesis of ^18^F-Labeled AAs via P–^18^F Bond Formation

**Figure 20:**
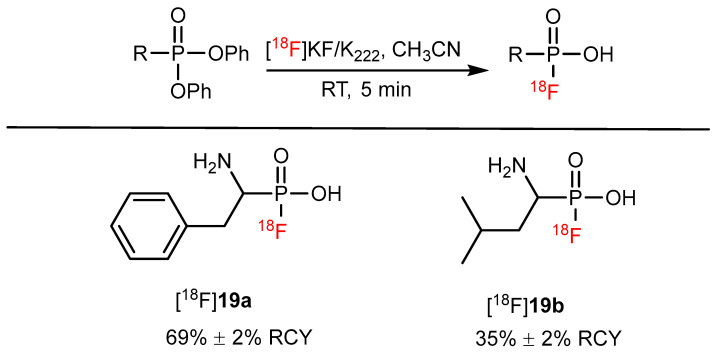
Synthesis of [^18^F]**19a** and [^18^F]**19b** via a nucleophilic substitution by F^−^ on phosphonate prostheses. Modified from Wang et al. [74].

In 2021, Li et al. reported an F^−^ site-specific nucleophilic substitution reaction on phosphonates that can be used for the one-step ^18^F-labeling of biomolecules containing common active groups. ^18^F-labeled AA mimics such as [^18^F]**19a** ([^18^F]PFA-Phe) and [^18^F]**19b** ([^18^F]PFA-Leu) have also been radiosynthesized in this way with RCYs of 69% and 35%, respectively (Figure 20) [74]. One of the limitations of this is that the similarity between fluorophosphonic acid and carboxylic acid needs further research.

## 10. Synthesis of ^18^F-Labeled AAs via S–^18^F Bond Formation

**Figure 21:**
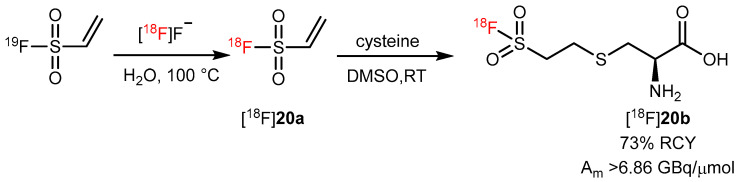
Radiosynthesis of [^18^F]Cys-ESF. Modified from Zhang et al. [77].

Ethenesulfonyl fluoride (ESF) can react with nucleophiles such as thiols, amines, and enamines to provide high yields and is considered to be one of the strongest Michael acceptors [75,76]. Therefore, [^18^F]**20a** ([^18^F]ESF) can be used as a potential prosthetic group to radiolabel AAs. AAs such as cysteine and tryptophan were successfully conjugated with [^18^F]**20a** to obtain yields between 39% and 73%. However, [^18^F]AA-ESFs showed low stability in rat serum over 2 h, and only [^18^F]**20b** had a purity of 12%. The other AA conjugates were completely defluorinated within 1 h (Figure 21) [77]. These findings suggest that S–F bond stability is greatly dependent on the conjugate. Thus, a radiosynthon that contains di-tert-butyl groups could potentially provide protection and prevent hydrolysis of the sulfur–[^18^F] fluorine bond [78].

**Figure 22:**
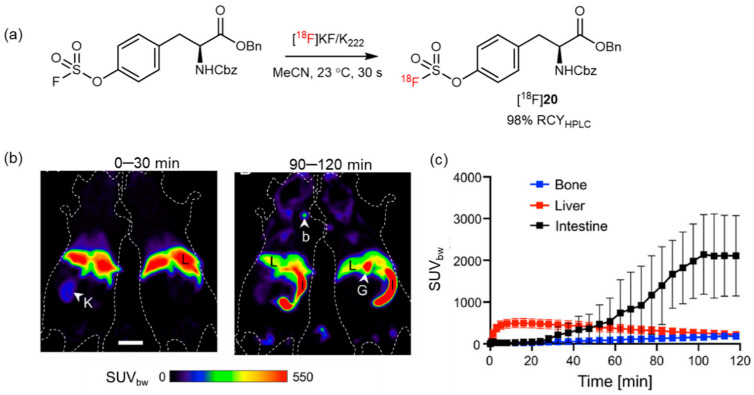
(**a**) Synthesis of [^18^F]**21** by a [^18^F]SuFEx reaction. Modified from Zheng et al. [79]. (**b**) Evaluation of [^18^F]**21** in healthy mice (*n* = 4) using micro-PET. (**c**) Time-activity curves demonstrated slow washout from the liver, high concentration in the intestine, and a low uptake in bone. Abbreviations: b—bone (humeral head); G—gall bladder; I—intestine; K—kidney; L—liver. Reprinted with permission from ref. [80], Copyright 2022 Elsevier Masson SAS.

Recently, an ultrafast isotopic exchange method has been reported to prepare aryl [^18^F]fluorosulfates by sulfur fluoride exchange (SuFEx) between aryl fluorosulfate and [^18^F]fluoride. This method has been used for the ^18^F-radiolabeling of Tyr analogs ([^18^F]**21**) with 98% RCY (Figure 22a) [79]. A study has reported the injection of [^18^F]**20** in healthy mice to determine its in vivo stability. A considerably low bone radioactivity uptake (maximum SUV_bw_ 155 ± 43, 90–120 min p.i.) indicated the high stability of [^18^F]**21** against in vivo defluorination (Figure 22b,c) [80]. Therefore, SuFEx radiofluorination shows potential to accelerate the development of novel AA PET tracers.

## 11. Synthesis of ^18^F-Labeled AAs via Si–^18^F Bond Formation

**Figure 23:**
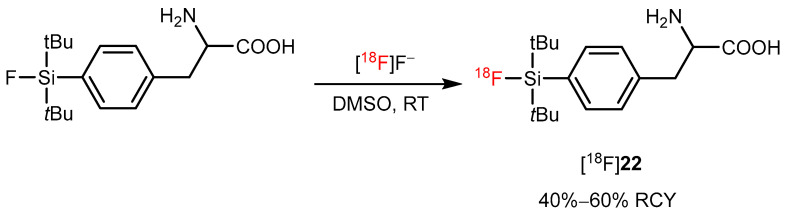
Synthesis of the [^18^F]**22**. Modified from Iovkova et al. [81].

Iovkov et al. radiosynthesized a silicon-based fluoride acceptor (SIFA)-modified phenylalanine through ^18^F/^19^F exchange reaction; however, the SiFA-modified AA (such as [^18^F]**22**) is not used for imaging AAT. It serves as a prosthetic group that incorporates into peptides and proteins for direct [^18^F]-fluoride labeling in the late stage (Figure 23) [81,82].

## 12. Conclusions and Perspectives

As AA PET imaging plays a vital role in metabolic molecular imaging, fast and convenient labeling methods are crucial. However, the ^18^F labeling of AAs is still challenging using traditional S_N_Ar or S_N_2 substitution reactions for the lack of reactive site. In addition, the complicated structure of the prosthetic group, which may impact the tracer’s bioactivity, hampers its wide utility. Thus, novel radiofluorination methods with short synthesis time and safe agents are urgently needed (Table 1). For example, CMRF is an important strategy that can label aromatic AAs in high yields; BAAs can serve as general imaging probes for AATs using a structure-based bioisosterism strategy. To make ^18^F-labeled AA probes more routine in clinical use, the “ideal” synthesis method could be optimized in the following aspects:

- Easy synthesis and high stability of the precursor;
- High regioselectivity and functional group tolerance (to avoid manipulation of the protecting group);
- Scale-up synthesis with automation module.

Given the continuous demand for novel AA PET radiopharmaceuticals in precision medicine, an ever-growing toolbox of radiofluorination methods is of importance to bridge the gap between the unmet clinical needs and the ongoing progress in modern fluorine-18 chemistry. Radiochemists are always searching for simplified radiochemical methods that are able to introduce the ^18^F radionuclide in a kit-like manner under mild conditions. Additional methods for the radiosynthesis of AA probes for clinical use are thus warranted.

## Figures and Tables

**Table 1:** Overview of new strategies for ^18^F-labeled amino acids’ radiosynthesis.

Strategy	^18^F Labeling Reaction Site	Example Radiotracer	Catalyst	Reaction Conditions	RCY	Advantages	Limitations	Ref.
CMRF	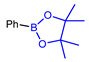	[^18^F]**2**	Cu(OTf)_2_(py)_4_	[^18^F]KF/K_222_, DMF, 110 °C, 20 min	55% ± 23%	High RCY; deprotection reaction was also carried out	Hard to perform on automation module	[35]
CMRF	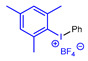	[^18^F]**4a**	(CH_3_CN)_4_CuOTf	[^18^F]KF/18-crown-6, DMF, 85 °C, 20 min	(23% ± 5% RCC, n = 3)	Moderate temperature (85 °C); high selectivity	The corresponding precursors are difficult to synthesize	[32]
CMRF	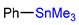	[^18^F]**5c**	Cu(OTf)_2_,	[^18^F]KF, pyridine, DMA, 140 °C, 5 min	56% ± 12% RCC	High functional group tolerance; high yield	Potential metal contamination	[42]
Ruthenium-mediated ^18^F-deoxyfluorination	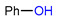	[^18^F]**7a**	CpRu(COD)Cl, *^ipr^*lmCl	[^18^F]F^−^, DMSO:MeCN = 1:1, 125 °C, 30 min	19% ± 4%	Scale-up synthesis with automation	Potential metal contamination	[44]
Manganese porphyrin mediated ^18^Flabeling strategy	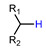	[^18^F]**8a**	Mn(TPFPP)OTs	[^18^F]F^−^, PhIO, K_2_CO_3_, CH3CN/acetone, 10 min, 50 °C	48% ± 5%	Without pre-activation in reaction site	Lack of automation synthesis study	[47]
Photo-aliphatic radiofluorination	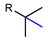	[^18^F]**9a**	NaDT	[^18^F]NFSI, hv (365 nm) H_2_O/CH_3_CN, 40 min	23% ± 3%	High regioselectivity	Relative low molar activity (<10 MBq/µmoL)	[50]
Photo-aromatic radiofluorination	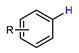	[^18^F]**11a**	10	[^18^F]NBu_4_, TEMPO, MeCN:t-BuOH = 4:1, 0 °C, 450 nm laser (3.5 W), 30 min, O_2_	5% ± 1%	Large substrate scope	Hard to perform on the automation module	[51]
Photoredox-catalyzed deoxyfluorination of phenol derivatives	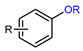	[^18^F]**15a**	14	[^18^F]TBAF, TBAHCO_3_ solution, DCE:tBuOH:MeCN = 3:4:1, 30 min, 0 °C, air/N_2_	42% ± 3%	High yield	Substrates with unprotected amines are unsuitable	[53]
Copper-mediated radio trifluoromethylation	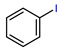 or 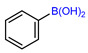	[^18^F]**16a**	CuBr or CuI	DMF, [^18^F]HCF_3_, 130 °C, 10 min; or DMF, [^18^F]CuCF_3_ air, 20 °C, 1 min	48% ± 7%	High molar activity (22 to 32 GBq/µmmol)	No PET imaging	[56]
Difluorocarbene-derived radio trifluoromethylthiolation	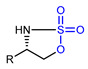	[^18^F]**17**	PDFA	[^18^F]KF/K_222_, S_8_ CH_3_CN, 70 °C, 5 min	14%	Adopt a structure-based bioisosterism strategy	Relatively low RCY (14% ± 3%)	[58]
Isotope exchange reaction based on B-^18/19^F bond	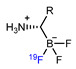	[^18^F]**18a**	-	[^18^F]F^−^, pH = 2.5 pyridazine buffer, 80 °C, 5 min	>60%	Efficient synthesis; high yield and molar activity	It is hard to separate precursor from product	[63]
Nucleophilic substitution reaction on phosphonates	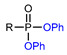	[^18^F]**19a**	-	[^18^F]KF/K_222_, CH_3_CN, RT, 5 min	69% ± 2%	High functional tolerance	No PET imaging	[74]
Ethenesulfonyl fluoride as prosthetic group	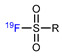	[^18^F]**20b**	-	[^18^F]F^−^, H_2_O, 100 °C	73%	An efficient way to label amino acids and proteins	Low stability in serum	[77]
Sulfur fluoride exchange reaction	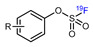	[^18^F]**21**	-	[^18^F]KF/K_222_, CH_3_CN, 23 °C, 30 s	99%	Ultrafast, late-stage labeling	The corresponding precursors are difficult to synthesize	[79]
Isotope exchange reaction based on Si-^18/19^F bond	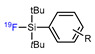	[^18^F]**22**	-	[^18^F]F^−^. DMSO, RT	40–60%	Stable in aqueous media; suitable for late-stage fluorination	Huge prosthetic group may interfere with functional groups of AA	[82]
